# Very low-calorie ketogenic diet (VLCKD): an antihypertensive nutritional approach

**DOI:** 10.1186/s12967-023-03956-4

**Published:** 2023-02-17

**Authors:** Luigi Barrea, Ludovica Verde, Pasquale Santangeli, Stefania Lucà, Annamaria Docimo, Silvia Savastano, Annamaria Colao, Giovanna Muscogiuri

**Affiliations:** 1 Dipartimento di Scienze Umanistiche, Università Telematica Pegaso, Via Porzio, Centro Direzionale, isola F2, 80143 Napoli, Italy; 2 Dipartimento di Medicina Clinica e Chirurgia, Unit of Endocrinology, Centro Italiano per la cura e il Benessere del paziente con Obesità (C.I.B.O), Federico II University Medical School of Naples, Via Sergio Pansini 5, 80131 Napoli, Italy; 3 Department of Public Health, University of Naples Federico II, Naples, Italy; 4 Department of Cardiovascular Medicine, Cleveland Clinic, Cleveland, OH USA; 5 Department of Medical and Surgical Sciences, University “Magna Graecia” of Catanzaro, 88100 Catanzaro, Italy; 6 Dipartimento di Medicina Clinica e Chirurgia, Unità di Endocrinologia, Diabetologia e Andrologia, Università degli Studi di Napoli Federico II, Via Sergio Pansini 5, 80131 Napoli, Italy; 7 Cattedra Unesco “Educazione alla Salute e Allo Sviluppo Sostenibile”, University Federico II, 80131 Napoli, Italy

**Keywords:** Very low-calorie ketogenic diet, VLCKD, Obesity, Hypertension, Blood pressure, Inflammation, Fat mass

## Abstract

**Background:**

Obesity is accompanied by hormonal, inflammatory and endothelial alterations. These alterations induce a stimulation of several other mechanisms that contribute to the hypertensive state and to increase the cardiovascular morbidity. This pilot, open - label, single- center, prospective clinical trial aimed to evaluate the effect of very low- calorie ketogenic diet (VLCKD) on blood pressure (BP) in women with of obesity and hypertension.

**Methods:**

A total of 137 women, who met the inclusion criteria and accepted to adhere to VLCKD, were consecutively enrolled. Assessment of anthropometric parameters (weight, height, and waist circumference), body composition (through bioelectrical impedance analysis), systolic (SBP) and diastolic blood pressure (DBP) and blood sample collection were carried out at baseline and after 45 days of the active phase of VLCKD.

**Results:**

After VLCKD all the women experienced a significant reduction in body weight and an overall improvement of body composition parameters. In addition, high sensitivity C reactive protein (hs- CRP) levels were significantly diminished (p < 0.001), while phase angle (PhA) increased by almost 9% (p < 0.001). Interestingly, both SBP and DBP were significantly improved (-12.89% and − 10.77%, respectively; p < 0.001). At baseline, SBP and DBP showed statistically significant correlations with body mass index (BMI), waist circumference, hs-CRP levels, PhA, total body water (TBW), extracellular water (ECW), Na / K ratio, and fat mass. Even after VLCKD, all correlations among SBP and DBP with the study variables were statistically significant, except for the association between DBP and Na / K ratio. Changes (%) in both SBP and DBP were associated with ∆BMI%, ∆PhA% and ∆hs- CRP levels (p < 0.001). In addition, only ∆SBP% was associated with ∆waist circumference (p = 0.017), ∆TBW (p = 0.017), and ∆fat mass (p < 0.001); while only ∆DBP% was associated with ∆ECW (p = 0.018), and ∆Na / K ratio (p = 0.048). After adjusting for ∆BMI, ∆WC, ∆PhA, ∆TBW, and ∆fat mass, the correlation between changes in ∆SBP and ∆hs -CRP levels remained statistically significant (p < 0.001). Similarly, the correlation between ∆DBP and ∆hs- CRP levels also remained statistically significant after adjustment for ∆BMI, ∆PhA, ∆Na / K ratio, and ∆ECW (p < 0.001). From multiple regression analysis ∆hs- CRP levels seemed to be the main predictor of changes of BP (p < 0.001).

**Conclusion:**

VLCKD reduces BP in women with of obesity and hypertension in a safely manner.

## Introduction

Cardiovascular diseases (CVD) continue to be the main cause of mortality and morbidity worldwide [1]. Despite major advances in diagnosis and treatment, the prevalence of CVD continues to increase in parallel with the growing prevalence of obesity and associated metabolic disorders [2]. Elevated blood pressure, even when not reaching defined cutoff levels that are diagnostic of hypertension, is a major risk factor for CVD including vascular injury, stroke, myocardial infarction, and heart failure [3]. Although the causes of primary hypertension are not completely understood, obesity appears to be a major culprit. Risk estimates from studies in multiple populations indicate that as much as 65–75% of the risk for primary hypertension can be attributed to weight excess and obesity [4]. However, the distribution of adipose tissue also be important in determining the impact of obesity on blood pressure and metabolic disorders such as dyslipidemia, insulin resistance, hyperinsulinemia, and type 2 diabetes mellitus [5].

Epidemiologic data consistently demonstrate a direct linear relationship between elevated body mass index (BMI) and blood pressure: as weight increases, blood pressure increases [6]. Correspondingly, the blood pressure lowering effect on weight loss also appears to be linear, with higher achieved weight loss resulting in greater decline in blood pressure [7, 8]. Meta-analyses of randomized controlled trials (RCTs) of lifestyle modifications (diet and exercise) demonstrate that every 1 kg of weight loss corresponds to a short-term (2–3 year) decline in systolic blood pressure (SBP) of 1 mmHg [7, 8]. A more recent study on the comparative efficacy of lifestyle modifications versus bariatric surgery showed that participants randomized to bariatric surgery took 20–30% less antihypertensive medication during the first four years of follow-up [9]. This difference is likely, at least in part, due to substantially greater weight loss observed individuals who underwent bariatric surgery compared to lifestyle modifications (mean 21.8% vs. 9.6% at five years) [9].

While bariatric surgery has proven to be a viable treatment option for the severe obesity and its complications, there is clearly a need for less invasive alternatives. Recent research has suggested that long-acting analogs of the gut hormone, glucagon-like peptide 1 (GLP-1) as an anti-obesity treatment [10]. Few years ago, liraglutide, a GLP-1 receptor agonist, was approved by the US Food and Drug Administration as an obesity treatment option and shown in clinical trials to be effective in reducing and sustaining body weight loss (3.0 mg / day) [11]. Moreover, although there are no published RCTs of liraglutide 3.0 mg / day specifically among patients with obesity and hypertension, it was associated with reduction in blood pressure in phase 3 trials among patients with obesity [12]. Furthermore, liraglutide’s cardiovascular safety has been demonstrated at the dose of 1.8 mg among patients with type 2 diabetes [13].

The combination drug therapy of naltrexone / bupropion is another anti -obesity drug option. However, in phase 3 trials, naltrexone / bupropion was associated with placebo adjusted increase in SBP and diastolic blood pressure (DBP) changes of + 1.5 mmHg and + 1.2 mmHg, respectively, at 1 year [14]. Blood pressure increases were greater at week 8 for naltrexone / bupropion with mean placebo adjusted SBP and DBP changes of + 2.4 mmHg and + 2.1 mmHg, respectively, suggesting that this combination drug therapy raises blood pressure to a greater degree early in treatment before weight loss has occurred. Furthermore, an ambulatory blood pressure monitoring sub study revealed that naltrexone / bupropion was associated with placebo-adjusted 24-hour average SBP and DBP changes of + 2.9 mmHg and + 3.0 mmHg, respectively, at 6 months and corresponding changes of + 2.6 mmHg and + 2.9 mmHg at 1 year, suggesting that elevation in 24-hour blood pressure persists over long-term exposure [14]. Unfortunately, after an inappropriate public disclosure of confidential interim data by the study sponsor, the naltrexone / bupropion cardiovascular outcomes trial was terminated early [15]; thus, the cardiovascular safety of this drug therapy remains uncertain.

Thus, unlike bariatric surgery, which is associated with a significant reduction in blood pressure and the use of antihypertensive drugs for most patients [14], anti-obesity drugs, while generally improving glycemic outcomes, did not produce a consistent and clinically significant improvement in blood pressure. In the context of obesity treatments, very low-calorie ketogenic diet (VLCKD) is becoming more and more a very promising nutritional intervention with high efficacy in terms of weight loss and improvement of metabolic parameters [16]: however, no data are reported on blood pressure changes. Thus, the aim of this prospective study was to investigate the effect of VLCKD on blood pressure (SBP and DBP) in a population of women with obesity and newly diagnosed hypertensive treatment naïve patients.

## Materials and methods

### Design and setting

This pilot, open-label, single-center, prospective clinical trial was initiated by the investigator to evaluate the effect of VLCKD on blood pressure in a population of women with obesity and newly diagnosed treatment-naïve hypertensive patients. Women were enlisted from May 2020 to March 2022 at the Unit of Endocrinology, Obesity Unit (*Centro Italiano per la cura e il Benessere del paziente con Obesità* “C.I.B.O.” and European Association for the Study of Obesity (EASO), Collaborating Centre for Obesity Management “EASO-COMs”), Clinical Medicine and Surgery Department, University of Naples Federico II, Naples, Italy. This clinical study has previously been approved by the Federico II Ethical Committee with protocol number 50 / 20. All women were informed of the study design and purpose, subsequently giving their informed consent.

### Population study

A total of 137 Caucasian women that had a history of failed dietary attempts and the desire to lose weight, were consecutively enrolled in this clinical study. The fundamental requirement was that the individuals to be enrolled were women patients with obesity and newly diagnosed hypertensive treatment naïve patients with blood pressure high normal and grade 1 hypertension, according to the 2018 ESC / ESH Guidelines for the management of arterial hypertension [17]. Inclusion criteria were: women aged 30–60 years, BMI ≥ 30 kg / m^2^, non-smokers, and willingness and ability to complete all research procedures. Exclusion criteria comprised patients with type 1 and type 2 diabetes mellitus, respiratory insufficiency, kidney failure and chronic kidney disease (estimated glomerular filtration rate < 60 mL / min/1.73 m^2^), hearth failure (NYHA III–IV), unstable angina, liver failure, a recent stroke or myocardial infarction (< 12 months), cardiac arrhythmias, atrio-ventricular block, unbalanced hypokalaemia, hypo-hyperthyroidism, active / severe infections, eating disorders and other severe mental illnesses, pregnancy and breastfeeding, alcohol and substance abuse; unable to give informed consent. In addition, women taking the following drugs were excluded from the study: hypertensive medication and beta-blockers, statins, corticosteroids, opioid pain medications, nonsteroidal anti-inflammatory drugs and drugs or dietary supplements known to affect weight and / or fat loss.

### Study protocol

**Fig. 1:**
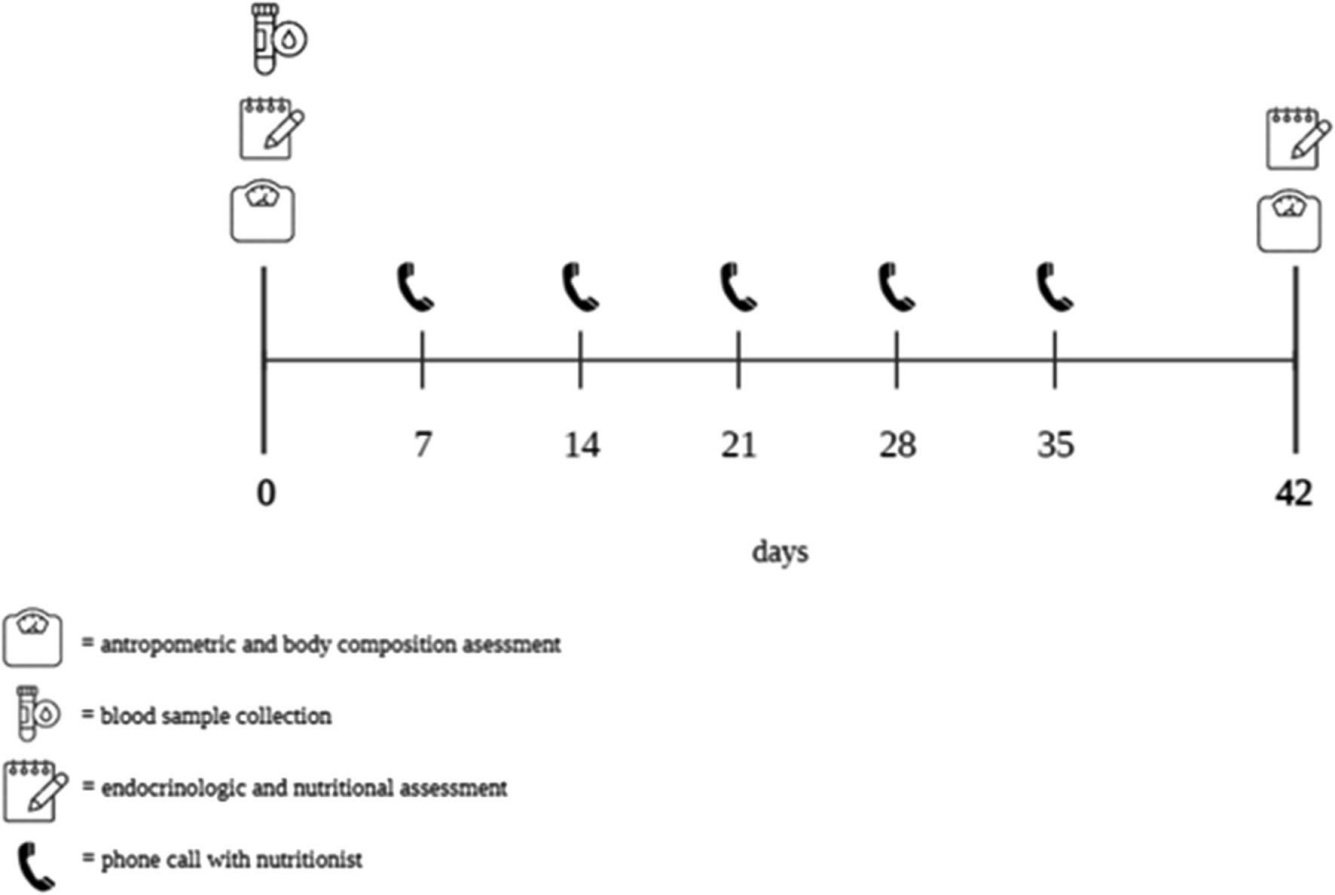
Timeline of assessment

At baseline (T0), all women underwent to an endocrinological and nutritional visit to evaluate the exclusion and inclusion criteria in the study (Fig. 1). When enrolled, an endocrinologist collected the complete clinical history and ruled out any contraindications to prescribing VLCKD, in accordance with the guidelines of EASO [16] and measured the blood pressure. Subsequently all women received nutritional counselling by the same qualified nutritionist which included the evaluation of anthropometric parameters (weight, height, and waist circumference), measurement of body composition and subsequent VLCKD protocol to be followed for 45 days. Follow-up visit were set up after 45 days during which all women underwent an endocrinological and nutritional visit (Fig. 1). Once a week, the nutritionist phone called the women to check the adherence to the nutritional protocols, recorded result measurement of ketone bodies from capillary blood. In addition, the nutritionist recorded any changes in physical activity levels and /or food and drink intake other than those in VLCKD protocol. Physical activity levels were assessed using a YES /NO response (at least 30 min /day aerobic exercise). Women were asked to maintain their current level of physical activity throughout the study (Fig. 1)

### Anthropometric measurements

Anthropometric measurements were performed by the same-trained health care operator at a time between 8 and 10 am. During the measurements, taken after an overnight fast, women wore light clothing and no shoes. Body weight was determined using a calibrated beam scale (Seca 711; Seca, Hamburg, Germany) to the nearest 0.1 kg while as well as height was measured using a wall -mounted stadiometer (Seca 711; Seca, Hamburg, Germany) to the nearest 0.5 cm. BMI was calculated as weight divided by the square of height [weight (kg)/height^2^(m^2^)]. According to BMI values, women were classified into overweight (25.0–29.9 kg / m^2^), grade I obesity (30.0–34.9 kg / m^2^), grade II obesity (35.0–39.9 kg / m^2^), and grade III obesity (≥ 40.0 kg / m^2^) [18]. Waist circumference (WC) was measured in women with uncovered waist having, standing with feet together, arms loosely along the sides, and breathing normally [19]. A nonelastic tape was used with an accuracy of 0.1 cm at the midpoint between the lowest costal bones and the highest point of the iliac crest. In women with the highest degree of obesity or where the narrowest point of the waist was not available, WC was assessed with 0.1 cm at the umbilical level [19].

### Bioelectrical impedance analysis (BIA)

Body composition was assessed by multifrequency bioelectrical impedance analysis (BIA 101 RJL with 800 A current and 50 kHz frequency, Akern Bioresearch, Florence, Italy) performed by the same nutritionist with the same BIA device to reduce variability between devices and between observers. The examination was performed according to the guidelines of the European Society of Parenteral and Enteral Nutrition (ESPEN) [20]. Specifically, during the BIA assessment, women were in the supine position with limbs slightly spaced from the body, removing shoes and socks and emptying their bladder 30 min earlier. They had not had any food or drink or exercise in the previous six hours, nor had they consumed alcohol 24 h before the examination. The contact surfaces were cleaned with alcohol immediately before electrode placement. Electrodes (BIATRODES Akern Srl; Florence, Italy) were placed on the right hand (proximal to the phalangeal-metacarpal joint) and the right foot (distal to the transverse arch). Sensor electrodes were placed on the right wrist (midway between the distal projection of the radius and ulna) and on the right ankle (between the medial and lateral malleoli). Every day, the qualified nutritionist checked the BIA device with resistors and capacitors of known value. Phase angle (PhA) in degrees (°) was calculated as arctangent reactance (X_c_) / resistance (R) (180/π) from the conditions at 50 kHz. Fat mass (FM), fat-free mass (FFM) and skeletal muscle mass (MM), expressed in kg, and total body water (TBW), intracellular water (ICW) and extracellular water (ECW), expressed in liters, were evaluated.

### Blood pressure assessment

SBP and DBP were measured in all women three times using a random zero sphygmomanometer (Gelman Hawksley Ltd., Sussex, UK) after 10 min sitting at rest, finally reporting the average of the last two values.

### VLCKD intervention

Women who fulfilled inclusion / exclusion criteria underwent VLCKD protocol with total meal replacement consisting of three main phases (active, re -education, and maintenance) [16]. A commercial weight  -loss program was used for VLCKD (New Penta Srl, Cuneo, Italy). The active phase diet was planned by the nutritionist and recommended by the endocrinologist. As for diet composition, total energy intake was < 800 kcal / day and it was provided by 13% carbohydrates (< 30 g / day), 43% protein (1.3 g / kg ideal body weight), and 44% fat. During VLCKD, replacement meals with high biological value were used, with protein coming from whey, soy, eggs, and peas. Supplementation of B-complex vitamins; vitamins C and E; minerals, including potassium, sodium, magnesium, and calcium; and omega-3 fatty acids at the same dosage was planned to maintain the physiological acid / base balance (PentaCal, New Penta, Ltd., Cuneo, Italy) [16].

### Compliance to VLCKD

Compliance to the recommendations for VLCKD and physical activity was assessed by weekly individual telephone counseling by an endocrinologist and nutritionist. In addition, once a week, women were asked to measure β-hydroxybutyrate from capillary blood using test strips (Optium Xceed Blood Glucose and Ketone Monitoring System; Abbott Laboratories, Chicago, IL, USA). More specifically, at baseline and at the end of the dietary intervention (day 45), β-hydroxybutyrate levels were measured by the nutritionist at the outpatient clinic. Furthermore, blood ketone levels were also checked directly by the women at home once a week, in the morning on an empty stomach, preferably at the same time, and the levels were reported to the nutritionist during the telephone interview, which as carried out once a week.

### Measurement of high sensitivity C reactive protein (hs-CRP) levels

Venous blood samples were collected in the morning between 8.00 and 10.00 am after an overnight fast (at least 8 h). Hs-CRP levels were assessed by a high-sensitivity nephelometric assay (CardioPhase hsCRP kit, Siemens Healthcare Diagnostics, Marburg, Germany). The lower limit of detection was 0.01 mg / L and the CV of intra- and interassay was < 7%. According to Centers for Disease Control and Prevention and the American Heart Association, based on baseline and 45 days of VLCKD hs-CRP levels, all women were further classified into three groups: low cardiovascular risk (< 1.0 mg / L), intermediate cardiovascular risk (1.0–3.0 mg / L), and high cardiovascular risk (≥ 3.0 mg / L) [21].

### Statistical analysis

Were included in this statistical analysis all women who completed the study, including all anthropometric measurements, blood pressure, inflammatory biomarker, and body composition parameters at baseline and after 45 days of VLCKD (active phase). These data were analyzed using MedCalc® package (Version 12.3.0 1993–2012 MedCalc Software bvba—MedCalc Software, Mariakerke, Belgium) and the IBM SPSS Statistics Software (PASW Version 21.0, SPSS Inc., Chicago, IL, USA). The Kolmogorov–Smirnov test was used to test data distribution. Skewed parameters were normalized by logarithm transformation and re-converted into tables and figures. The mean ± standard deviation (SD) or percentages (%) were used to present the data. The outcomes between the baseline and after 45 days of VLCKD (active phase), in particular weight, BMI, WC, hs-CRP levels, PhA, TBW, ECW, Na / K ratio, FM, SBP, and DBP were compared using the Student’s paired *t*-test. The chi square (χ2) test was used to test differences in frequency distribution across categories of physical activity, BMI, and hs-CRP levels. Pearson’s correlation was used to assess the association among SBP and DBP with anthropometric measurements, inflammatory biomarker, and body composition parameters of the study population at baseline and after 45 days of VLCKD (active phase). In addition, Pearson’s correlation also been used for assessing the association among percentage changes (delta ∆%) pre / post intervention of study parameters (∆BMI, ∆WC, ∆hs-CRP levels, ∆PhA, ∆TBW, ∆ECW, ∆Na / K ratio, and ∆FM). Two partial correlations were performed to adjust the associations among ∆SBP and ∆DBP with ∆hs -CRP levels for confounding factors (∆BMI, ∆WC, ∆PhA, ∆TBW, ∆Na / K ratio, ∆ECW, and ∆FM). Finally, two multiple linear regression analysis models (stepwise method), expressed as R^2^, Beta (β) and *t*, with the SBP and DBP as a dependent variable (Model 1 and Model 2, respectively) were used to estimate the predictive value of anthropometric measurements, inflammatory biomarker, and body composition parameters. Variables with a variance inflation factor (VIF) > 10 were excluded to avoid multicollinearity. The *p*-values below 5% were considered statistically significant. Because this was a pilot study, no power calculations were performed. Therefore, all findings need to be confirmed by larger clinical trials.

## Results

A total of 137 women with obesity, aged 46.48 ± 10.19 years, met the inclusion / exclusion criteria, were included in these statistical analyses. All women were evaluated at baseline and after 45 days of VLCKD (active phase). Adherence to VLCKD was assessed and confirmed in all women by a qualified nutritionist through a telephone interview once a week. In detail, all women were called the day before the interview to instruct them to do the ketosis capillary test and the results were registered on the day of the interview by the nutritionist. In addition, patients were asked about their levels of physical activity. No patients changed their physical activity levels as required at the baseline visit (43, 31.4% vs. 43, 31.4%).

Table 1 reported anthropometric measurements, blood pressure, inflammatory biomarker, and body composition parameters of the study population at baseline and after 45 days of VLCKD (active phase). All anthropometric measurements, BIA parameters, and hs-CRP levels were significantly diminished (p < 0.001), except for PhA, which increased by almost 9% (p < 0.001). Therefore, the distribution of women across BMI categories were significantly changed, with the decrease in the prevalence of grade III obesity (− 15.4%, p = 0.002), while the prevalence of overweight women was increased (+ 19.7%, p < 0.001). Similarly, after 45 days of VLCKD (active phase), the number of women at high cardiovascular risk decreased (-34.3%, p < 0.001), and the number of women at low cardiovascular risk increased (+ 19.0, p < 0.001); Table 1.

**Table 1:** Anthropometric measurements, inflammatory biomarker, and body composition parameters at baseline and after active phase

Parameters	Women at baseline *n* = 137	Women after VLCKD *n* = 137	∆%	* p-value
Weight (kg)	97.74 ± 14.23	90.77 ± 13.45	− 7.12	**< 0.001**
BMI (kg / m ^2^)	37.00 ± 4.47	34.37 ± 4.29		**< 0.001**
Overweight	–	27, 19.7	+ 19.7	χ^2^ = 27.77, ***p*** **< 0.001**
Grade I obesity	51, 37.2	54, 39.4	+ 2.2	χ^2^ = 0.06, *p* = 0.804
Grade II obesity	50, 36.5	41, 29.9	− 6.6	χ^2^ = 1.05, *p* = 0.305
Grade III obesity	36, 26.3	15, 10.9	− 15.4	χ^2^ = 9.64, ***p*** **= 0.002**
WC (cm)	108.93 ± 12.89	102.71 ± 12.50	− 5.63	**< 0.001**
Inflammatory biomarker				
hs-CRP levels (mg / L)	3.82 ± 4.19	2.07 ± 2.73	− 38.66	**< 0.001**
Low risk	25, 18.2	51, 37.2	+ 19.0	χ^2^ = 11.38, ***p*** **< 0.001**
Intermediate risk	44, 32.1	65, 47.4	+ 15.3	χ^2^ = 9.04, ***p*** **= 0.014**
High risk	68, 49.6	21, 15.3	− 34.3	χ^2^ = 35.21, ***p*** **< 0.001**
BIA parameters				
PhA (°)	5.43 ± 0.85	5.87 ± 0.87	+ 8.96	**< 0.001**
TBW (Lt)	40.83 ± 5.05	39.89 ± 5.05	− 2.21	**< 0.001**
ECW (Lt)	19.93 ± 2.94	18.61 ± 2.75	− 6.43	**< 0.001**
Na / K ratio	0.94 ± 0.13	0.92 ± 0.12	− 1.68	**0.011**
FM (kg)	43.49 ± 11.63	37.04 ± 10.50	− 14.73	**< 0.001**
Blood pressure				
SBP (mmHg)	140.88 ± 8.99	122.56 ± 10.08	− 12.89	**< 0.001**
DBP (mmHg)	88.90 ± 6.71	78.94 ± 6.68	− 10.93	**< 0.001**

Data are expressed as number and percentage (n, %) or mean ± standard deviation (mean ± SD) * A *p* value in bold type denotes a significant difference (*p* < 0.05)

*SD* standard deviation,* VLCKD* very low -calorie ketogenic diet,* BMI* body mass index,* WC* waist circumference,* hs-CRP* high - sensitivity C-reactive protein,* BIA* bioelectrical impedance analysis,* PhA* phase angle,* TBW* total body water,* ECW* Extracellular water,* Na* sodium,* K* potassium,* FM* fat mass,* SBP* systolic blood pressure,* DBP* diastolic blood pressure

**Fig. 2:**
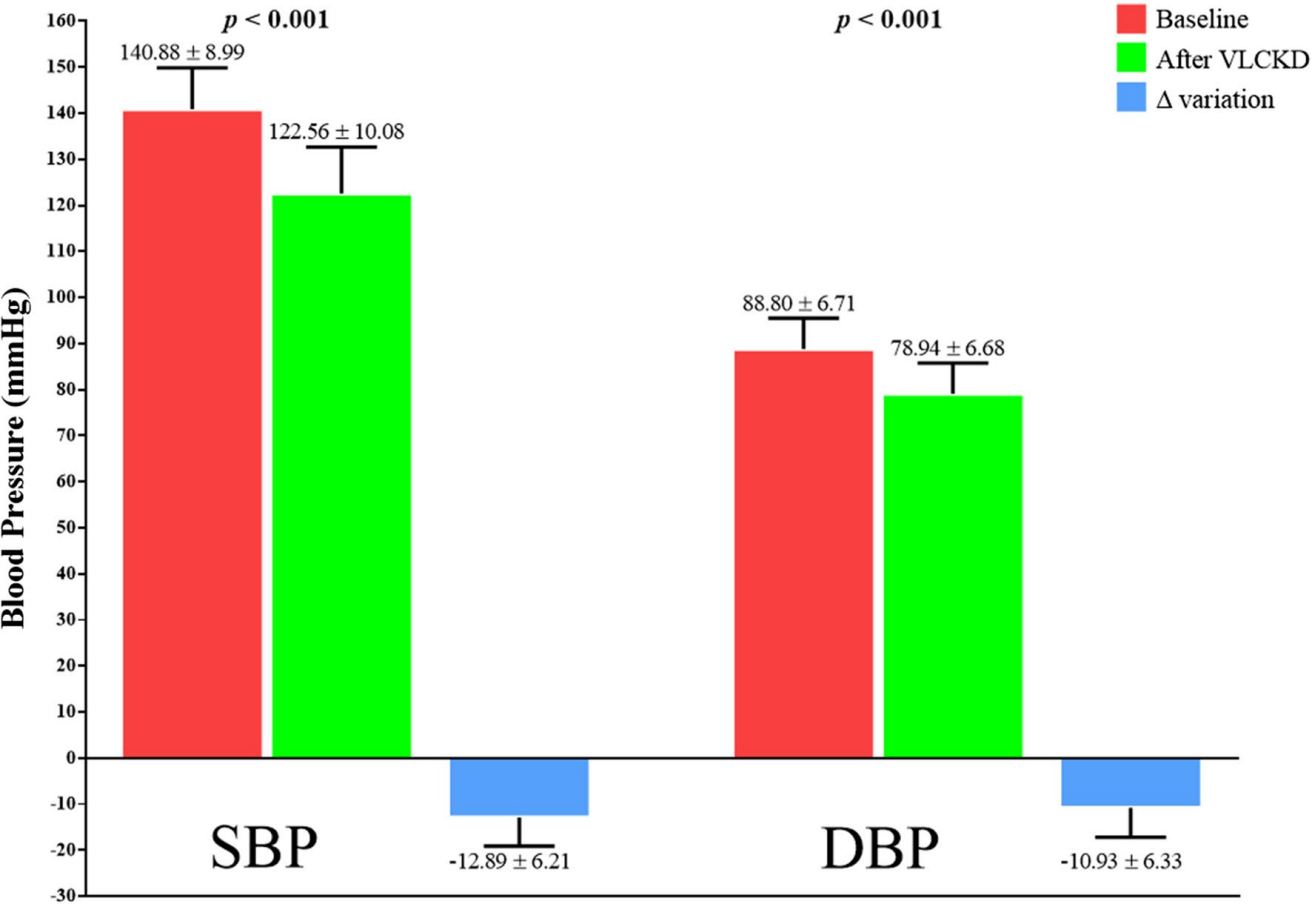
The change in blood pressure during the 45 days of VLCKD (active phase). After VLCKD, both SBP and DBP parameters were significantly decreased (p < 0.001). *VLCKD* very low- calorie ketogenic diet, *SBP* systolic blood pressure, *DBP* diastolic blood pressure

Interestingly, as showed in Fig. 2, after 45 days of VLCKD (active phase), both SBP and DBP were significantly improved (− 12.89% and − 10.77%, respectively; p < 0.001).

Table 2 showed the correlations among SBP and DBP with anthropometric measurements, inflammatory biomarker, and body composition parameters of the study population at baseline and after 45 days of VLCKD (active phase). At baseline, except for age, SBP and DBP showed statistically significant correlations with all study variables. Even after 45 days of VLCKD (active phase), all correlations among SBP and DBP with the study variables were statistically significant, except for the association between DBP and Na / K ratio, which maintains only a trend of significance (p = 0.055).

**Table 2:** The correlation among SBP and DBP with anthropometric measurements, inflammatory biomarker, and body composition parameters

Parameters	Baseline				After 45 days of VLCKD			
	SBP (mmHg)		DBP (mmHg)		SBP (mmHg)		DBP (mmHg)	
	r	* p -value	r	* p -value	r	* p -value	r	* p -value
Age (years)	0.060	0.484	− 0.059	0.492	0.006	0.943	− 0.117	0.175
BMI (kg / m^2^)	0.552	**< 0.001**	0.336	**< 0.001**	0.384	**< 0.001**	0.294	**< 0.001**
WC (cm)	0.497	**< 0.001**	0.317	**< 0.001**	0.367	**< 0.001**	0.277	**0.001**
hs - CRP (mg / L)	0.395	**< 0.001**	0.225	**0.008**	0.391	**< 0.001**	0.251	**0.003**
PhA (°)	− 0.374	**< 0.001**	− 0.222	**0.009**	− 0.383	**< 0.001**	− 0.232	**0.006**
TBW (Lt)	0.630	**< 0.001**	0.318	**< 0.001**	0.323	**< 0.001**	0.278	**0.001**
ECW (Lt)	0.767	**< 0.001**	0.418	**< 0.001**	0.509	**< 0.001**	0.377	**< 0.001**
Na / K	0.390	**< 0.001**	0.239	**0.005**	0.274	**0.001**	0.158	0.055
FM (kg)	0.546	**< 0.001**	0.288	**0.001**	0.359	**< 0.001**	0.314	**< 0.001**

* A *p* value in bold type denotes a significant difference (*p* < 0.05)

*SBP* systolic blood pressure,* DBP* diastolic blood pressure,* BMI* body mass index,* WC* waist circumference,* hs- CRP,* high -sensitivity C-reactive protein,* PhA* phase angle,* TBW* total body water,* ECW* Extracellular water,* Na* sodium,* K* potassium,* FM* fat mass

The correlation among changes in ∆SBP% and ∆DBP% with changes in anthropometric measurements, inflammatory biomarker, and body composition parameters were reported in Table 3. Changes in both ∆SBP% and ∆DBP% were associated with ∆BMI% and ∆PhA% and very strongly with ∆hs - CRP levels (p < 0.001). In addition, only ∆SBP% was associated with ∆WC (p = 0.017), ∆TBW (p = 0.017), and ∆FM (p < 0.001); while only ∆DBP% was associated with ∆ECW (p = 0.018), and ∆Na / K ratio (p = 0.048).

**Table 3:** The correlation among changes in ∆SBP% and ∆DBP% with changes in anthropometric measurements, inflammatory biomarker, and body composition parameters

Parameters	∆SBP		∆DBP	
	r	* p-value	r	* p-value
Age	− 0.047	0.586	− 0.075	0.383
∆BMI	0.352	**< 0.001**	0.168	**0.050**
∆WC	0.204	**0.017**	0.020	0.815
∆hs - CRP	0.621	**< 0.001**	0.452	**< 0.001**
∆PhA	− 0.301	**< 0.001**	− 0.204	**0.017**
∆TBW	− 0.204	**0.017**	− 0.014	0.868
∆ECW	0.155	0.071	0.202	**0.018**
∆Na / K	0.047	0.583	0.169	**0.048**
∆FM	0.373	**< 0.001**	0.126	0.143

* A *p* value in bold type denotes a significant difference (*p* < 0.05)

*SBP* systolic blood pressure,* DBP* diastolic blood pressure,* BMI* body mass index,* WC* waist circumference,* hs - CRP* high-sensitivity C-reactive protein,* PhA* phase angle,* TBW* total body water,* ECW* Extracellular water,* Na* sodium,* K* potassium,* FM* fat mass.

**Fig. 3:**
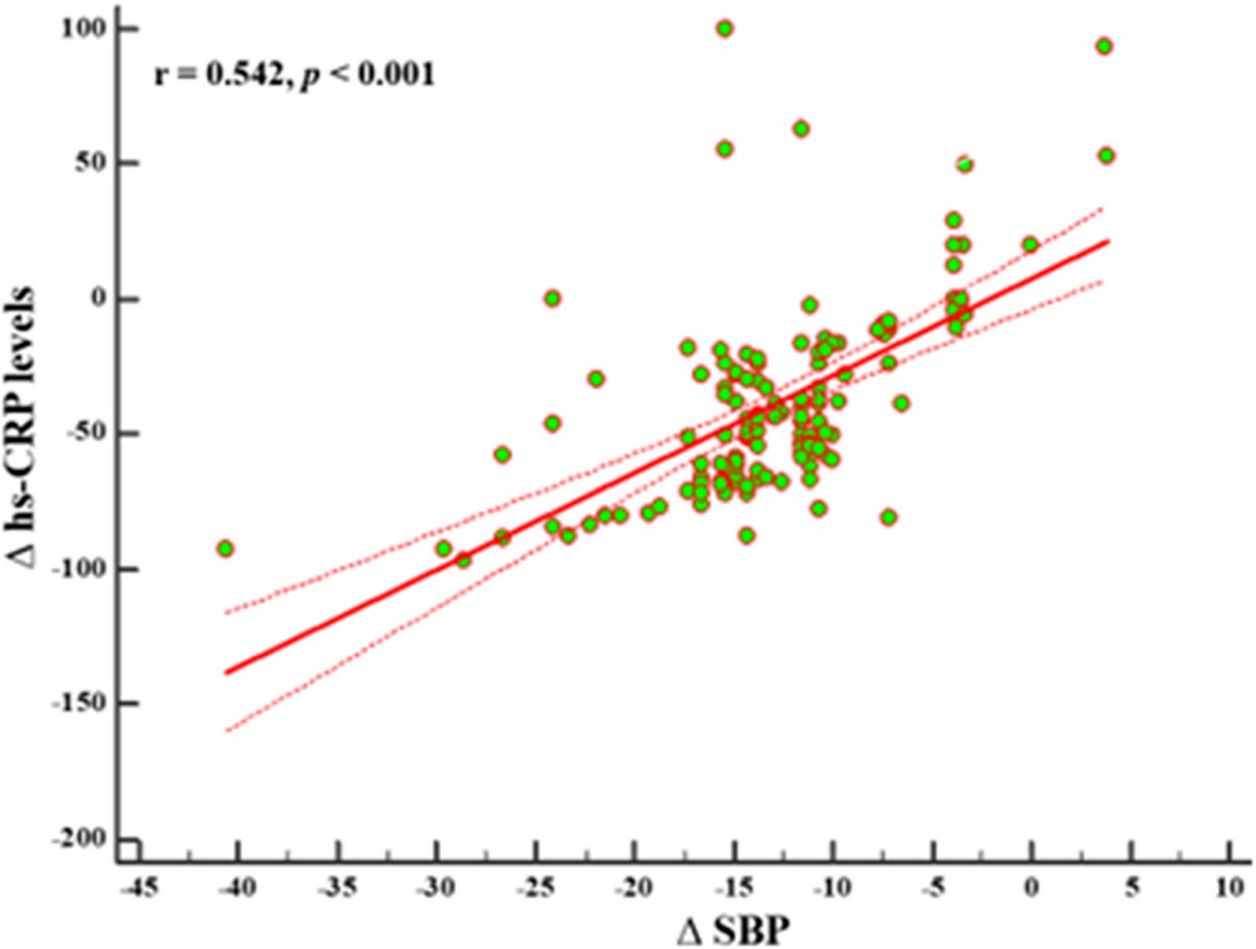
Correlation between changes in ∆SBP and ∆hs - CRP levels after adjusting for ∆BMI, ∆WC, ∆PhA, ∆TBW, and ∆FM. A p value in bold type denotes a significant difference (p < 0.05). *SBP* systolic blood pressure, *hs -CRP* high -sensitivity C-reactive protein

As reported in Fig. 3, after adjusting for ∆BMI, ∆WC, ∆PhA, ∆TBW, and ∆FM, the correlation between changes in ∆SBP and ∆hs - CRP levels remained statistically significant (r *=* 0.542, p < 0.001).

**Fig. 4:**
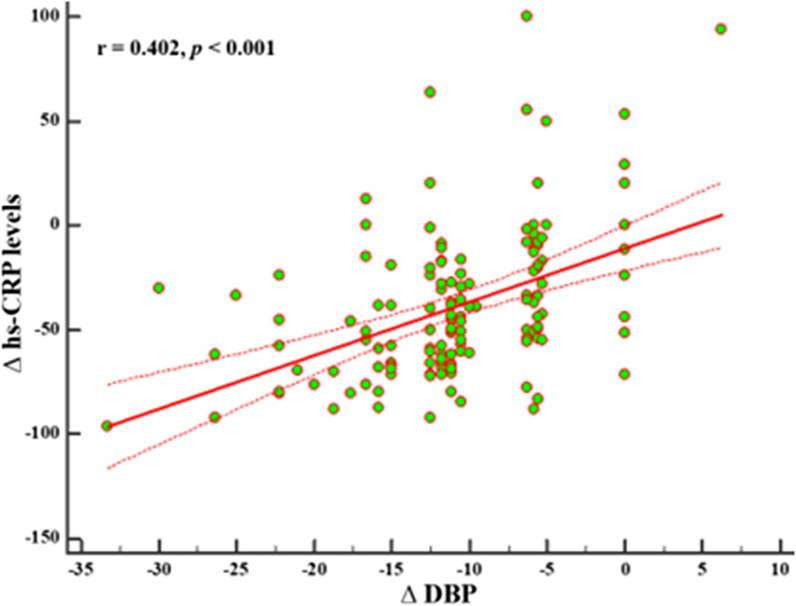
Correlation between changes in ∆DBP and ∆hs - CRP levels after adjusting for ∆BMI, ∆PhA, ∆Na / K ratio, and ∆ECW. A p value in bold type denotes a significant difference (p < 0.05). *DBP* diastolic blood pressure, *hs-CRP* high-sensitivity C-reactive protein

Similarly, the correlation between ∆DBP and ∆hs -CRP levels also remained statistically significant after adjustment for ∆BMI, ∆PhA, ∆Na / K ratio, and ∆ECW (r = 0.402, p = 0.001), Fig. 4.

To compare the relative predictive power of ∆BMI, ∆WC, ∆hs - CRP levels, ∆PhA, ∆TBW, and ∆FM associated with ∆SBP, we performed a multiple regression analysis (Model 1), while to compare the relative predictive power of ∆BMI, ∆hs - CRP levels, ∆PhA, ∆Na / K ratio, and ∆ECW associated with ∆DBP, we performed a second multiple regression analysis showed as Model 2. In the first model, ∆hs - CRP levels were entered at the first step (p < 0.001), followed by the ∆FM (p < 0.001). In the second model, only ∆hs - CRP levels were entered at the first step (p < 0.001); Table 4.

**Table 4:** Multiple regression analysis models (stepwise method) with the SBP and DBP as a dependent variable (Model 1 and Model 2, respectively) to estimate the predictive value of anthropometric measurements, inflammatory biomarker, and body composition parameters

Parameters	Multiple Regression analysis			
	R^2^	β	*t*	*p-value
Model 1- SBP				
∆hs - CRP levels	0.381	0.621	9.21	**< 0.001**
∆FM	0.418	0.210	3.07	**0.003**
Variables excluded: ∆BMI, ∆WC, ∆PhA, and ∆TBW				
Model 2—DBP				
∆hs-CRP levels	0.199	0.452	5.89	**< 0.001**
Variables excluded: ∆BMI, ∆PhA, ∆Na / K ratio, and ∆ECW				

* A *p* value in bold type denotes a significant difference (*p* < 0.05).

*SBP* systolic blood pressure, *hs - CRP* high - sensitivity C - reactive protein, *FM* fat mass, *BMI* body mass index, *WC* waist circumference, *PhA* phase angle, *TBW* total body water, *DBP* diastolic blood pressure, *Na* sodium, *K* potassium, *ECW* Extracellular water.

## Discussion

To our knowledge, this is the first study to provide information on the effects of VLCKD on blood pressure in women with obesity and hypertension. Not surprisingly, all the women experienced a significant reduction in body weight and an overall improvement of body composition after 45 days of the active phase of VLCKD. As a new result we reported that VLCKD reduced blood pressure after a short period of active phase in women with obesity and hypertension. In particular, we found that SBP and DBP decreased by -12.89% and − 10.93%, respectively. This result seems particularly promising since, in addition to confirming the safety and efficacy of VLCKD as a tool for managing obesity, no other dietary intervention reported in the literature to date seems to achieve the same results in such a short time. Regarding the currently most widely used nutritional approach for the treatment of hypertension, the Dietary Approaches to Stop Hypertension (DASH), this produces significant reductions in blood pressure (− 5.2 mmHg for SBP and − 2.60 mmHg for DBP) after an average of 8 weeks of intervention [22]. The other most widely used nutritional intervention for managing obesity is the Mediterranean diet (MD). However, intervention studies have shown mild effects of the MD on blood pressure that require a long period of treatment and are likely a consequence of weight loss [23, 24]. In particular, the PREDIMED (PREvención con DIeta MEDiterránea) study compared the MD with a low - fat control diet in 7447 subjects with overweight or obesity and with more than 80% of subjects with hypertension [25]. After a follow - up period of 4 years, the PREDIMED study showed no change in SBP in both groups, while DBP decreased by 1.5- and 0.7 - mmHg in the MD intervention groups of extra virgin olive oil and mixed nuts, respectively [25].

Interestingly, at the end of the active phase of VLCKD, the levels of low - grade inflammation were statistically reduced in our women, and it was evidenced by significantly lower and higher hs - CRP levels and PhA, respectively. Moreover, at baseline and after 45 days, both SBP and DBP values correlated with hs - CRP levels, body weight and body composition parameters. The percentage change in blood pressure was also significantly correlated with the percentage changes in hs - CRP levels, PhA, and BMI. The positive association between SBP and DBP with hs - CRP levels were still evident after adjusting for confounding variables, in particular the percentage of weight loss. Finally, the reduction in inflammatory state induced by VLCKD was the main predictor of changes in blood pressure, including SBP and DBP after 45 days of VLCKD. As well - known inflammation contributes to the pathogenesis of hypertension [26]; indeed, the long - term inflammatory process, a common feature of obesity, increases the production of reactive oxygen species, causing oxidative stress that leads to endothelial dysfunction, i.e. the loss of normal tone and structure of blood vessels [27]. In addition, when inflammation is prolonged, the bioavailability of nitric oxide decreases, interrupting its main function as a vasodilator, so that relaxation and vasodilation of the blood vessels are absent [27]. In particular, hs - CRP, the prototypical acute - phase reactant, is not just an innocent bystander in the increase in blood pressure, but rather an active factor through several mechanisms: these include suppression of nitric oxide and increased generation of reactive oxygen species, as well as increased release of endothelin - 1 and interleukin - 6 from endothelial cells, molecules that contribute to vascular stiffness and thus may promote the development of hypertension [28]. As expected, biomarkers of inflammation, including hs - CRP and various cytokines are elevated in subjects with hypertension [29] and perhaps of even more importance, multiple cohorts have now identified hs - CRP as a predictor of incident hypertension in subjects without hypertension at baseline [30–35]. Furthermore, the reduction of PhA, a BIA - derived parameter that reflects the integrity and size of cell membranes, has been associated with inflammatory markers [36, 37], and previous clinical studies have reported that PhA could be a screening tool to identify inflammation in subjects with obesity and several other inflammation - based diseases [38–41]. We had already demonstrated in a previous uncontrolled, single - center, open - label pilot clinical study in 260 women with overweight or obesity that hs - CRP levels decreased (∆-38.9 ± 45.6%) while PhA increased (∆+8.6 ± 12.5%) after 31 days of an active phase of VLCKD [42]. Therefore, we can speculate that the reduction of inflammation levels could also have beneficial effects on blood pressure levels and that VLCKD, being able to accomplish the aforementioned, could be among the indispensable tools for the prevention and management of hypertension in obesity.

Finally, we found significant reductions in TBW (− 2.21%), ECW (-6.43%), Na / K ratio (-1.68%) and this led us to hypothesize that changes in body water compartments observed at the end of the active phase of VLCKD may have contributed to the observed changes in blood pressure. To our knowledge this is the first study evaluating the relation between body water compartments and blood pressure in women with obesity and hypertension. In this regard, we found out positive correlations of SBP and DBP values with BIA analysis parameters and, among other, statistically significant correlations with TBW, ECW and Na / K ratio. In agreement with our findings, in a study of 72 children to assess the relationship between body water compartments (assessed by BIA) and blood pressure, SBP and DPB correlated significantly with TBW and ECW [43]. We know that TBW, ECW and the Na / K ratio are correlated with volaemia [44]. A sufficiently significant decrease in ECW (in parallel with a reduction in the Na / K ratio and TBW) causes a reduction in effective circulating blood volume, which in turn can lead to reductions in blood pressure [45]. Vice versa, an association between high ECW and high blood pressure has been reported in the literature [46–48] and several explanations have been proposed: (a) high ECW increases venous return, which in turn increases cardiac output and vascular resistance [49]; (b) subjects with a high ECW have a higher salt intake, which is associated with high blood pressure [49]; (c) compensatory mechanisms of chronic dehydration may play a role, such as abnormal activation of the renin-angiotensin-aldosterone system, dysfunction of the Na / K pump and dysregulation of the sympathetic nervous system [50].

We certainly want to mention some limitations of our study. Firstly, the design of our study did not include a control arm so we cannot establish with certainty the superiority of VLCKD in reducing blood pressure compared to other dietary interventions. However, we found no data in the literature superior to ours on the same outcome with other dietary interventions. Second, we assessed blood pressure using a random zero sphygmomanometer, an approach that has limitations due to poor reproducibility, observer, and patient variability and the ‘white coat’ effect [51]. In future, it would be necessary to confirm these results by 24 - hour ambulatory blood pressure monitoring, the current gold standard for measuring blood pressure [52]. Finally, we have no data on the heart rate and cannot rule out a possible compensatory tachycardia to the reduction in volaemia. However, such a compensatory tachycardia is not likely to counterbalance the antihypertensive effects following VLCKD.

## Conclusion

**Fig. 5:**
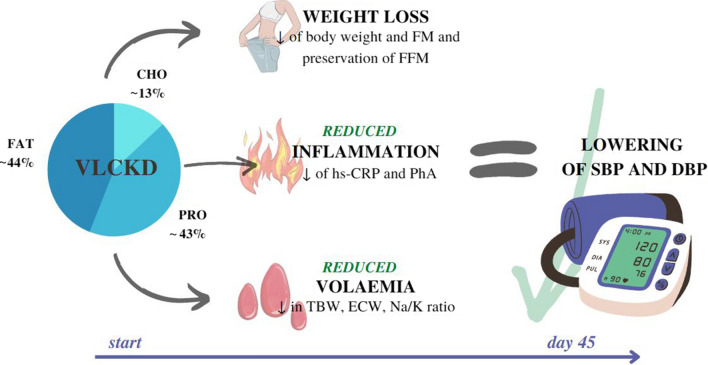
Very low-calorie ketogenic Diet mechanisms underlying blood pressure reduction

 The study provides the first evidence of safety and efficacy of VLCKD in the management of obesity and hypertension. Due to its beneficial metabolic and anti-inflammatory effects (Fig. 5), VLCKD should be considered a safe and effective intervention strategy in appropriately selected and motivated patients suffering from obesity and hypertension, which can lead to a reduction, or even discontinuation, of drug therapy, potentially leading to disease remission.

## Data Availability

The datasets used and / or analyzed during the current study are available from the corresponding author on reasonable request.

## Abbreviations

CVD: Cardiovascular diseases
BMI: Body mass index
RCT: Randomized controlled trial
SBP: Systolic blood pressure
DBP: Diastolic blood pressure
VLCKD: Very low-calorie ketogenic diet
WC: Waist circumference
BIA: Bioelectrical impedance analysis
PhA: Phase angle
Xc: Reactance
FM: Fat mass
FFM: Fat- free mass
MM: Muscle mass
TBW: Total body water
ICW: Intracellular water
ECW: Extracellular water
hs -CRP: High sensitivity C reactive protein
SD: Standard deviation
VIF: Variance inflation factor
DASH: Dietary Approaches to Stop Hypertension
MD: Mediterranean diet
